# Effect of Infodemic Regarding the Illegal Sale of Medications on the Internet: Evaluation of Demand and Online Availability of Ivermectin during the COVID-19 Pandemic

**DOI:** 10.3390/ijerph18147475

**Published:** 2021-07-13

**Authors:** András Fittler, Latifat Adeniye, Zoltán Katz, Richárd Bella

**Affiliations:** 1 Faculty of Pharmacy, Department of Pharmaceutics, University of Pécs, Rókus Street 2, 7624 Pécs, Hungary; adeniyelatifah@yahoo.com (L.A.); bella.richard@pte.hu (R.B.); 2 Department of Operational Medicine, University of Pécs, Medical School, Szigeti Street 12, 7624 Pécs, Hungary; zoltan.katz@aok.pte.hu

**Keywords:** illegal online pharmacies, infodemic, coronavirus, ivermectin, self-medication, misinformation

## Abstract

The COVID-19 pandemic and the related infodemic generated confusion and increased demand of various pharmaceuticals, ushering in the opportunity for illicit online vendors to fill a gap in the marketplace using potentially dangerous products. The aim of our study is to provide evidence regarding increased demand, online availability and consumer accessibility of ivermectin, an anthelmintic agent, without substantiated indications in reference to SARS-CoV-2. In our study, we combined infodemiology methodology aligned with search engine result assessment and website analytics to evaluate patient safety risks. Users’ Google queries regarding ivermectin were trending and peaked during the last week of November 2020 and March 2021. Consumers more likely found links leading directly or indirectly (via redirection) to illegal online retailers representing nearly half (53.3%) of search engine result links regarding the first three result pages in December 2020 and topped off at 73.3% by March 2021. Illicit medicine retailers outnumbered and outranked their legitimate counterparts and dominated the first search engine results page. A vast majority (77.7%) of the identified online pharmacies were characteristically rogue; more than half (55.5%) offered prescription-only products without a valid medical prescription. Our results illustrate connection between infodemic and its consequences on the illicit online pharmacy market

## 1. Introduction

The online pharmacy market has undergone significant growth during the past two decades as society is shifting to a more digitalized world. Admittedly, motivations for purchasing medicines or healthcare products online include an abundance of information, vast product assortment, cost savings, anonymity, convenience and home delivery [1,2]. There are two broader categories of online vendors offering medications: legitimate internet pharmacy websites providing high-quality pharmacy services and illicit online sellers, which unfortunately outnumber their legitimate competition [3,4]. Meanwhile, it evolved into a vehicle for counterfeit vendors since the online space ideally conceals the identity of illegitimate sellers and easily confuses consumers [5]. Although rather contradictory, potential “additional benefits” can be perceived by individuals regarding the illegal online pharmacy market: direct purchasing of prescription-only medicines (POM) without consultation, accessibility of limited or no availability products (controlled drugs, unapproved generics, recalled medications or those with off-label recommendations), discrete payment options using cryptocurrency and lastly, no maximum purchase quantity [3,4,6,7,8].

In parallel, the rising “self-prescribing culture” creates the continuously increasing demand regarding POMs sales through illegitimate vendors [5]. Evidently, numerous health risks (e.g., misinformation by unreliable health claims inconsistent with evidence-based medicine guidelines, side effects, disease progression and drug abuse) resulting in “digital iatrogenesis” and financial concerns (including payment insecurity or customs seizure) may arise [2,4,9].

The European Medicines Agency (EMA) has called public attention to the potential threats posed by unregistered websites, emphasizing unauthorized vendors intend to exploit fears and concerns during the ongoing pandemic by offering dubious products to treat or prevent COVID-19 [10]. In the US, the FDA has issued warning letters to website operators engaged in illegal activity and marketing products rife with fraudulent COVID-19 prevention and treatment claims [11]. Although, various authorities and agencies alerted patients and the public to avoid self-diagnosis and medication, the online and uncontrolled accessibility of unapproved medications against the coronavirus disease seemingly are unavoidable.

Although previous infectious disease outbreaks (e.g., the severe acute respiratory syndrome (SARS) epidemic, H1N1 pandemic and Middle East respiratory syndrome (MERS) epidemic) have also been registered in past decades, only the announcement of COVID-19 pandemic led to panic buying, stockpiling, scarcity and increased cost of daily supplies, medicines and protective equipment around the globe [12,13]. To cite an example, the first COVID-19 lockdown was associated with a significant increase in weekly purchase of public pharmacies from wholesalers in Germany, in March 2020 [14]. Similarly, the online pharmaceutical- and health-product market was affected by the changing perspectives of patients towards purchasing medicines online, also driven by panic buying and increased product demands [12,15,16]. In addition, falsified agents thrive on shortages giving the opportunity for illegal actors to fill the gap upon the market by potentially dangerous products [17].

With the help of technology, novel medical research results were disseminated far more efficiently. Simultaneously, the plethora of coronavirus-related information, including mis- and disinformation may undermine the public health response [18]. Health authorities have long since recognized how rumor, stigma and conspiracy theories as emerging threats to the preparedness and control measures of public health emergencies swirl with rapid velocity influencing society. The term “infodemic” refers to the overabundance of information posing concerns to the public to distinguish fact from fiction [19]. This phenomenon has the potential to decrease the trust of community regarding health agencies and influence communication among health care professionals and emergency responders [20].

Until vaccinations were initiated, professional and public focus was on potential forms of drug treatment. Promising drug candidates for COVID-19 treatment were identified by the repurposing of existing drugs approved for other diseases with good safety profiles and potential antiviral activity. As of mid-April 2021, a sum of 9149 clinical trials have been registered related to COVID-19 by the World Health Organization’s (WHO) International Clinical Trials Registry Platform [21], while ClinicalTrials.gov database lists 5359 clinical studies related to COVID-19 [22]. Despite various agents and drugs currently undergoing investigation in support of the treatment of COVID-19, as depicted in clinical trials around the globe, only the antiviral agent remdesivir is approved by the Food and Drug Administration (FDA) for the treatment of COVID-19 [23], and there is lack of evidence to make definitive conclusions regarding safe and effective treatment options as of April 2021. Consequently, there is continuous confusion regarding the proven and unproven effectiveness of various active ingredients, guidelines are updated regularly and several “miracle treatment” options are discussed in the news and social media platforms, often with conflicting conclusions.

Preliminary findings including preclinical data regarding the potential effects of various medications (e.g., hydroxychloroquine, azithromycin, ivermectin, etc., whether independently dispensed or in combination with others) without substantiated indications for COVID-19 were clearly hyped leading to increased demand, stockpiling, self-medication [24], escalation of drug-shortages [25,26], reports of hospitalization [27], illegal online availability [28], false claims and falsified medicine sales [17,19,29].

Based on the information acquired by the Airport Directorate of the National Tax and Customs Administration of Hungary, as of mid-December 2020, many consignments—which to the best of our knowledge, originated from Serbia—with the same content, were intended to be posted from Hungary. The suspicious shipments were confiscated during the last week of November 2020, and the first two weeks of December 2020. A total of 54 packages containing 630 boxes of ivermectin, in the form of 12 mg tablets (20 pcs/pack, labeled as “Iverlast” Kachhela), equivalent to approximately 12,700 tablets were seized by custom officials. Ivermectin is an antiparasitic drug widely used for decades in animals and humans, approved in various countries for the treatment of intestinal strongyloidiasis and onchocerciasis. During the pandemic, several sources claimed ivermectin to be effective in higher dosages for both prophylaxis and in the treatment of COVID-19, despite the lack of meaningful evidence for clinical activity or clinical efficacy in patients afflicted with COVID-19. Meanwhile, guidelines could not support such claims, and the originator’s corporate statement recommended against the use of ivermectin in the treatment of COVID-19 [23,30,31]. It must be noted, similarly to other countries, the active substance is not used for systemic treatment in human medicine within Hungary, and marketing authorization of the oral preparation was not available at the time of our study. As we have hypothesized, ivermectin was inevitably offered for retail use online in which we sought to identify its potential origin, labeling and health claims.

## 2. Aims

In our study, we aim to provide evidence of increased demand, illegal online availability and consumer accessibility of ivermectin using a multiple approach method. Further, to reveal online sources of ivermectin products by evaluating specific marketing approaches, promotional messages, and the characteristics and health claims of online vendors’ sites in a representative sample of search engine results over a span of four consecutive months. Consequently, we aim to illustrate a direct link between infodemic and its consequences regarding the illicit online pharmacy market.

## 3. Methods

In our study we combined infoveillance methodology (Google search trends, relevant national news articles) with our previously published risk-based method for online medicinal products. Such a complex risk assessment method allows the evaluation of the probability of the online purchase of ivermectin products (by search engine result (SER) assessment) and the severity of patient safety risks associated with internet procurement opportunities (via website analytics) [32,33].

### 3.1. Evaluation of Search Trends and Assessment of Triggering News

We assessed the dynamics of national internet searches and potential triggering news related to the application of ivermectin throughout Hungary during the COVID-19 pandemic, beginning in January 2020 and extending through March 2021. The relative search volume for the term “ivermectin” for Hungary were downloaded from Google Trends (accessed on 16 April 2021) [34] and plotted against national coronavirus case data available from WHO confirmed cases of COVID-19 [35]. The statistical correlation was calculated using Pearson correlation to explore the relationship between Google Trends data (weekly relative search popularity) and WHO COVID-19 data (weekly confirmed COVID cases throughout Hungary). Data was analyzed using SPSS Statistics 26 for Windows. Relevant Hungarian online news articles were identified using a Google News aggregator (accessed on 16 April 2021) [36], specifically using the term “ivermectin”. Next, news articles were manually categorized by the authors described as either “potentially triggering” or “averting”.

### 3.2. Obtaining and Evaluation of Search Engine Results

To find evidence for the correlation between online availability and accessibility of a product, Google, the most widely used internet search engine in the country, was used with English search term, “buy ivermectin online”. Interestingly, Hungarian language searches yielded no relevant results. We aimed to simulate users’ search behavior and to find potential websites they most likely visit when searching for ivermectin. The first organic (non-sponsored) 30 SERs were evaluated in monthly intervals beginning in December 2020 and extending through March 2021, during the last week of each month. Our sample represents a vast majority of individuals’ manual searches, as studies on Google organic search ranking click through rate (CTR) behavior shows, majority (approx. 85%) of individuals do not scroll past the first page, while results regarding the second page have a CTR of less than 1% for each position [37]. Documented search result data includes the following: date, uniform resource locator (URL), domain name, SER ranking, final destination website URL for redirections and website category. The prevalence of website categories within Google’s TOP10 SER links were sub-analyzed. Following the manual evaluation of links, final destination websites were categorized as: legitimate online pharmacies; illegal medicine retailers (rogue online pharmacies); compromised sites redirecting to international illegal medicine retailers; global B2C or B2B e-commerce websites (e.g., amazon.com, indiamart.com); price comparison or intermediary pages; telemedicine sites; veterinary online pharmacies; informative websites (e.g., news, authority, medicine database); and websites not accessible at the time of evaluation (e.g., 404 error). Only websites offering medicine for retail use were included in our study for additionally detailed content evaluation.

### 3.3. Content Evaluation of Websites Offering Ivermectin for Retail Use

Indicators of potential threats in patient safety were determined based on website legitimacy, distributional characteristics (drug availability without a valid prescription, brand name and cost) and information regarding indications/health-claims. The legitimacy of websites offering medicines for retail use were determined using LegitScript.com internet pharmacy verification database, and a site was labeled as “rogue” if it does not meet LegitScript Internet pharmacy verification standards. Additional indicia of valid online pharmacy operations were assessed, and an online pharmacy was categorized as “certified” if we found evidence for national accreditation logos indicating valid online pharmacy operations. The possibility of purchasing products was determined by the opportunity to place selected products in the cart; however, no product purchase was made. Availability of contact information of the vendor (street address, telephone number and e-mail), prescription requirement, health claims and information regarding ivermectin products was assessed based on the information provided on the landing page from SERs, main page, Contact Us and FAQ pages. Potential indications of use (e.g., recommendation for coronavirus disease) was assessed using manual evaluation of the product description section.

## 4. Results

### 4.1. Correlation of Google Trends and WHO Confirmed COVID-19 Cases, Potentially Triggering News

The analysis of news articles yielded 18 pieces of news published from 25 November extending through the 25 March, approximately one third of these (*n* = 7) were within the first two weeks of the above time period. Ten (55.6%) articles were categorized as averting, while the remainder as potentially triggering (e.g., updates of ongoing clinical trials, approval for use in a neighboring country). It must be noted, first and presumably, the most triggering news regarding ivermectin was entitled, “With a Nobel Prize-winning drug against the coronavirus?” posted by one of the largest Hungarian news portals on 25 November 2020. However, it was deleted shortly after its publication, making it inaccessible for our study.

**Figure 1:**
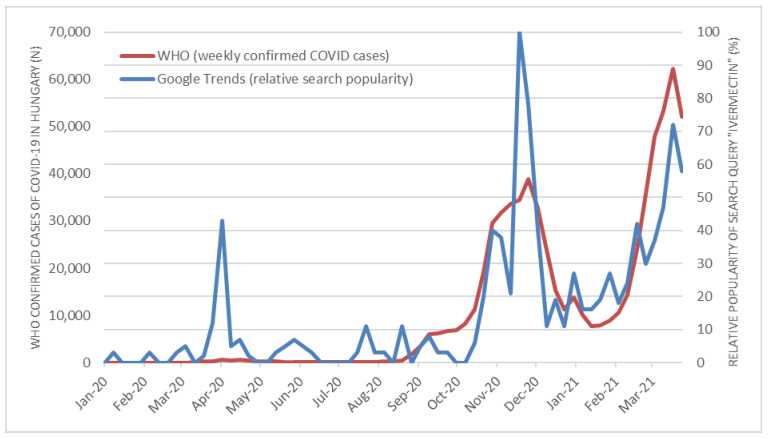
Trend analysis of infoveillance metrics.

Actual users’ Google queries regarding the active ingredient was trending and peaked during the last week of November 2020 and March 2021. At the same time, the second and third wave of the COVID-19 pandemic has resulted in a surge of confirmed cases throughout Hungary (see Figure 1). Time series data regarding online search behavior associated with the term “ivermectin” and national COVID-19 data on cases indicate statistically significant correlation according to Pearson correlation analysis (R value = 0.827, *p* ≤ 0.01). Most likely the increased threat caused by the large number of new confirmed cases and the confusion generated by the media regarding the potential beneficial effects of ivermectin have contributed to the high level of interest, and presumably the demand for the active ingredient.

### 4.2. Search Engine Results for Ivermectin Beginning in December 2020 and Extending through March 2021

Repeated monthly evaluation of links within the SER landscape provides an insight regarding the reactions of the online pharmacy market supply side for the increased search volume for ivermectin. We have hypothesized consumer search and navigation behavior is affected by three key factors: (a) relevance (content of the title- and meta-description appearing in SER), (b) ranking (position within SER pages) and (c) probability of occurrence (relative number of links within SER pages referring to a final destination website). Accordingly, these were considered as potential indicators of searchers’ click tendency. Links were considered relevant if the content indicated the website offered pharmaceuticals for retail use direct to consumers.

As we used buyer-specific keywords, the majority of the links were relevant during the study period. Most (63.3–83.3%) referred searchers to online pharmacy websites offering human medicine direct to consumers. While only a few links led to B2C or B2B e-commerce websites (e.g., indiamart.com or amazon.com), but never amongst the Google’s TOP10 search engine results. A link to a veterinary online pharmacy appeared only on the third SER page in December 2020 and January 2021.

Legitimate online pharmacies (verified by LegitScript.com international, or pharmacyregulation.org national database) accounted for only 3–10% of the 30 evaluated search results. In contrast, consumers were more likely to find links leading directly or indirectly (via redirection) to illegal medicine retailers representing nearly half (53.3%) of SER links of the first three result pages in December 2020 and reaching 73.3% by March 2021. Although search engines typically refer consumers to relevant online resources quickly, when ivermectin related search terms were issued, we distinctly observed search–redirection attacks [38,39,40] regarding compromised high-ranking websites with a seemingly unrelated domain name and redirection of users to illegal online pharmacies. In the case of search–redirection attacks, in reference to the SER page, users are redirected through a redirection chain to an illegal website. The chain consists of a landing page on the compromised website (e.g., paris.edu/search/Buy+Ivermectin+Online...), one or more intermediate page(s) (e.g., MayoClinic.shop), and finally, users are redirected to the final destination online pharmacy website (e.g., anonympharmacy.com). As Table 1 illustrates, the number of websites affected by search–redirection attacks dynamically evolved over the four-month period and hacked links significantly outnumbered direct “traditional” SER links of rogue online pharmacies.

The remaining website categories (price comparison, telemedicine and informative websites) were considered non-relevant, as we found no opportunity to purchase medicinal products directly. Informative content was published by authorities (EMA, FDA) and news portals alerting the public of potentially fraudulent COVID-19 products and the unapproved uses for ivermectin, or providing medical information (e.g., WebMD). Links leading to HTTP status code 404 “not found error” pages were not accessible at the time of our evaluation. Although we could not evaluate website content or potential redirection, this status likely indicates hacked content was identified by attentive website administrators.

The prevalence of website categories in Google’s TOP10 search engine results is less diverse. Although in February, one legitimate internet pharmacy (healthwarehouse.com) ranked #9 amongst the most valuable SERs, in which a majority of TOP10 links belonged to compromised sites redirecting visitors to international illegal medicine retailers (90%, 60%, 100% and 100% for December, January, February and March, respectively).

Search engine rank position has a significant impact upon CTR behavior. In Table 2**,** we calculated the average search engine rank position of organic links of website categories, identified each month and the relative standard deviation (RSD) of rank numbers. Low average rank number indicates better position on SER pages and presumably higher number of visitors. RSD depicts the distribution of rankings, consequently, a higher percentage indicating scattered positions in SER pages. Links of legitimate online pharmacies appeared on the second and first SER page in December and January, respectively; however during February and March, their positions dropped to the third SER page, supposedly resulting in marginal CTR. Strikingly, as presented in Table 1, deceptive and illegal medicine retailers far outnumber and outrank legitimate pharmacists and their sheer numbers dominate the first search engine result page. Many rogue retailers are well cloaked, concealed and masters regarding cyber disguise, or spoofing. Links to all other website categories also lag behind, suggesting lower CTR and consequently, a small number of visits to these sites.

### 4.3. Websites Offering Ivermectin for Retail Use

During the four-month interval, a total number of 120 SER links were evaluated with 92 relevant links offering ivermectin for retail use. Excluding duplicate links, three global B2C or B2B e-commerce websites (aliexpress.com, amazon.com and dir.indiamart.com), one veterinary online pharmacy (homelabvet.com) not selling human-intended medication, we identified and evaluated 20 internet pharmacy websites. A majority of the websites appeared only once in SERs during the time of our study (min.: 1, mode: 1), however, some were linked directly or via redirection regularly (max.: 26). Two registered online pharmacies from the UK appeared within SERs; however, ivermectin was not listed or the product link has been removed from the site by the end of our study period, thus no further investigation was performed. Website characteristics of the remaining 18 internet pharmacies are summarized in the Appendix A.

Although customers have the opportunity to contact customer service through contact forms on the websites, a street address was not highlighted in 10 (55.5%) sites. In several cases (e.g., anonympharmacy.com, dr-cheap.com, trustnetpharmacy.com), we observed contact phone number matches indicating partnership or affiliated networks. None of the sites revealed both street address and the telephone number of corporate entities. The possibility of information exchange among healthcare professionals (e.g., online chat, phone, online form, etc.) was available in six (33.3%) sites. Based on LegitScript’s legitimacy database, a vast majority (*n* = 14, 77.7%) of the online pharmacies were rogue, three certified (16.6%) and one (5.5%) was not in the database. More than half of the online medicine vendors offered prescription-only products without a valid medical prescription (*n* = 10, 55.5%), while the remainder required previous medical consultation and valid prescription prior to placing an order.

**Figure 2:**
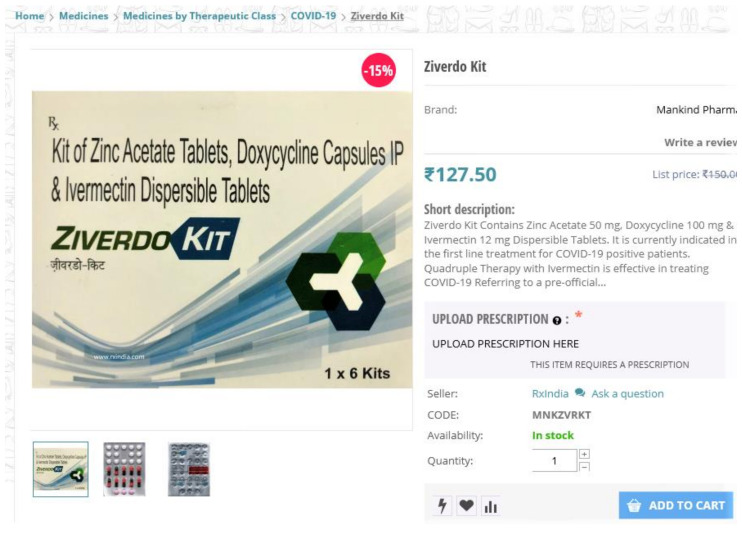
Website offering combination therapy containing ivermectin for the treatment of COVID-19 patients.

Available dosage strength ranged from 3 mg–12 mg/tablet. The smallest quantity offered for retail use was 1 tablet per pack, while consumers were offered large quantities in 12 mg tablets in a 180 pack. A greater number of websites (*n* = 17, 94.4%) displayed anthelmintic (treatment of parasitic infections) as a health claim in support of ivermectin products without any referral to coronavirus disease. On the other hand, in December 2020, a Ziverdo Kit offering “Quadruple Therapy” as the first line treatment for COVID-19 positive patients was offered for retail use by rxindia.com. The kit is stated to contain ivermectin 12 mg, doxycycline 100 mg and zinc acetate 50 mg (see Figure 2).

Ivermectin tablets were branded as, “Stromectol”, “Generic Stromectol” “Ivermectol” and Ivecop. Once again, “Iverlast” tablets were discovered while evaluating customs seizures and interestingly, were not listed on any website. Since we intended to determine an online source of the medication, we managed to perform a direct search on Facebook at the beginning (December 2020) and at the end (March 2021) of the timeline of our research using “ivermectin” and “IVM” search terms.

Assessments of search results were made based on the name and description of Facebook groups and whether the application approval required any sensitive or personal data. In December 2020, our search resulted in discovering a Facebook group for professionals entitled, “IVERMECTIN MD TEAM”. In our investigation of this group, including more than 7000 members, we could not only find information regarding therapy; however, imagery of its product line interestingly matched with packages earlier revealed by the staff of customs clearance. Since belonging to this group, we were not asked to present any proof nor substantiate we are professionals during the approval process regarding our application. Inexplicably, during a Facebook search performed in March 2021, the previously observed professional group could no longer be found. This finding factually supports how Facebook has heavily censored Ivermectin-related content during the span of time regarding our study [41]. By the end of our study period, the only adequate and relevantly popular group entitled, “LONG-HAULERS ON IVERMECTIN AND BEYOND” focused on the general public in the sharing their ivermectin therapy related experiences. Conversations in the group primarily expressed taking other medications and supplements during ivermectin therapy. Since our application was approved, and no proof nor substantiating was required in seeking ivermectin, it clearly shows how individuals can also join who are merely curious regarding Ivermectin therapy. In this group, no signs of direct marketing nor advertisement were present, and no posts or comments were found in which individuals openly described how they acquired their medicine. This currently available group seems to have no overlap with the former professional group.

## 5. Discussion

In response to COVID-19 pandemic, restrictive rules have been introduced to mandate social distancing in nearly all countries, worldwide. The lockdown has accelerated digitalization in every aspect of our lives and limited the access to services, including access to health-services. Moreover, the pandemic drove individuals into panicking, and it led to most individuals acquiring health-related information and even medical interventions from non-professional resources, including families and friends, the internet and social media. Consequently, crowds of nonconversant service users appeared online. The media has a crucial role in the widespread dissemination of information in referencing encouraging results of potentially beneficial therapies. These candidates came to focus in the already existing self-medication practice, in spite of the absence of effective, safe and proven medicine(s) against COVID-19. However, authorities informed the public, in which unproven therapies and substandard medical products may exacerbate the current public health emergency, yet individuals persist and are still searching for and using such ingredients such as ivermectin. The demand for unproven and non-substantiated drugs quickly escalates and are clandestinely marketed by illicit medicine retailers launching online pharmacy websites.

Ivermectin is an ideal directional molecule in many ways to illustrate the correlation of infodemic and the illegitimate online pharmaceutical landscape. As on the one hand, this inexpensive active ingredient has been used large scale for a long time. Ivermectin is one of the most widely used drugs for roundworm infections in developing countries, with which millions of people have been treated and therapeutic success has been achieved. The relatively recent awarding of the Nobel Prize for its discovery [42] further enhanced its recognition. Therapeutic benefits of the drug include its favorable side effect profile; however, it should be noted, its amount in the main indication of ivermectin is significantly lower than the amount recommended for COVID-19. Already at the time of the pandemic, observations published in professional journals about the antiviral effect were available. However, ivermectin became the focus of the attention when Australian researchers published the possibility of high dose ivermectin could stop the virus from replicating in cells [43,44]. Therefore, the professional and—due to the infodemic—the lay literature refers to ivermectin as a medicine expected to help patients in a life-threatening illness, which cannot be treated with any currently authorized medicine. As shown in our paper, ivermectin has been included in the treatment of coronavirus disease in many countries around the world, including off-label and compassionate use of the active substance. Such methods allow the use of unauthorized drugs under strict conditions for patients not included in clinical trials. In addition, at the time of writing, nearly 70 clinical trials are underway with the drug based on the Clinicaltrials.gov database [22]. Finally, ivermectin is authorized as a prescription only medicine, restricting access to the drug within the legal drug supply chain and consequently can direct patients to alternative sources of supply.

We found evidence regarding individual and affiliate networks of rogue retailers offering prescription-only ivermectin drugs which gained media attention during the second and third wave of the pandemic (December 2020–March 2021). Trends in Google searches using the expression, “buy ivermectin online” and the increasing number of relevant links among SER pages suggest an infodemic sparked panic purchasing of ivermectin. Search engine results were dominated by links leading visitors (by redirection or referral) to established network sites of illegitimate online pharmacies. Distinctly, the largest share (77.7%) of the online pharmacies were rogue, according to LegitScript database, and more than half (55.5%) of the online medicine vendors identified in our study sold medicine without a valid medical prescription.

Illegal actors embedded in the online pharmaceutical market, including rogue networks, are taking advantage of the pandemic by offering prescription-only ivermectin products with unproven efficacy against the coronavirus disease for retail use. Although, in most instances, we were unable to find direct referral to indications against COVID-19 on evaluated websites, it is highly probable retailers are capitalizing in on the pandemic. We have documented an unapproved health claim, indicating ivermectin as potential treatment for coronavirus disease in a fixed-dosage combination, marketed as the “Ziverdo Kit”. Often-referred to COVID kits are already available, despite the set of remedies combined drugs (e.g., hydroxychloroquine, azithromycin, ivermectin, anticoagulants, paracetamol and doxycycline) having no proven efficacy in COVID-19 therapy nor prevention [45]. In addition to the false sense of security, individuals may face the consequences of adverse effects of such medications. For example, in Brazil, at least five patients afflicted with COVID-19 succumbed due to drug-induced hepatitis after using a COVID kit [45]. The use of one or more of unproven drugs in the prevention or treatment of COVID-19 clearly bears a serious health threat. Possible side effects often associated with ivermectin include skin rash, nausea, vomiting, diarrhea, stomach pain, sudden drop in blood pressure, facial and limb swelling, neurological adverse events and liver injury [46].

Distinctly, ivermectin was authorized in limited use in the treatment of COVID-19 in several countries (e.g., South Africa and Slovakia in January 2021 [41,47]), while widely used throughout Latin America [42]). However, the WHO advised the drug should only be used within clinical trials to treat COVID-19 [48]. Based on the latest evidence regarding ivermectin, the European Medicines Agency (EMA) has concluded, available data does not support its use in the prevention and treatment of COVID-19. Clinical studies were contradictory regarding the benefits of ivermectin, consequently, the authority emphasized the need of further controlled clinical trials and the increased risk of side effects associated with higher doses. Thus, its use is not currently recommended [49].

Future research should focus on the online availability of additional unapproved treatments against COVID19. Further, test purchase of medications from illicit online vendors would provide valuable real-world data on the product quality and potential safety issues.

### Limitations and Strengths

The primary limitation regarding our study is the complete risk-based safety mapping was not performed, as the medications offered for retail use online were not purchased, and the qualitative or quantitative analysis of the products not conducted by the authors. The correlation of Google Trends data for “ivermectin” and a number of national confirmed COVID-19 cases have been evaluated; however, the influence of relevant online news articles was not included in our statistical analysis. Admittedly, the focus of our research is seemingly relatively narrow, due to the 4-month timeframe and search trends focusing on Hungary. However, national and international search trends and COVID-19 cases are comparable; moreover, SER page links and evaluated websites were international. Accordingly, our findings provide a wider context for unregulated prescription drug distribution and motivations for self-medication generally.

At the same time, we relied upon a dual approach method including infoveillance methodology, providing evidence for increased interest (suggesting demand) and increasing number of relevant links to online vendors of ivermectin in SERs (suggesting supply). Although, our study focuses only on ivermectin as an “exemplar” study medication, the authors believe similar mechanisms will manifest themselves regarding other ingredients affected by confusion during the pandemic. Furthermore, this active ingredient is ideal in illustrating the effects of infodemic upon the illegal online pharmacy market due to its repositioning, off-label use and reckless lack in prescription requirements.

## 6. Conclusions

The issues related to misinformation, self-medication and unregulated online pharmacies were evident for decades; however, the extent of the pandemic and parallel infodemic demonstrates more clearly than ever, the extent of public health risks resulting from digital iatrogenesis [9] and a trade-off between easy accessibility of scientific information and the premature public use of unproven medical interventions [28].

The pandemic has caused changes regarding the demand and access to medications and have facilitated self-care and self-medication behaviors among the public worldwide. General confusion, an infodemic and the lack of effective treatment seemingly attributed to individuals to repurpose various medications on the market without approved indications for COVID-19 as potentially effective treatments or prevention measures. In conclusion, several factors, e.g., prescription requirement, necessity of off-label approval by drug authorities or limitation of supply to prevent drug shortages, prohibit the general population to acquire such products from traditional and legitimate resources, hence, consumers turn to the illegitimate online pharmaceutical market.

Such behaviors and medical misinformation bear potentially devastating consequences for individuals (e.g., adverse effects, incorrect diagnosis, ineffective treatment, use of counterfeit and low-quality products) and health systems (e.g., hospital admissions, increased expenses, shortages and the spread of the virus). Consequently, online accessibility of unapproved treatments against COVID19 must be regularly monitored and websites offering such products without valid medical prescription for retail use must be regulated, made inaccessible by authorities; and censored by search engines and domain registrars. However, challenges can only be effectively managed by focusing on the demand side: consumers and patients. Pro-actively and frequently updated information provided by medical/pharmaceutical associations and regulatory bodies are critically important in fighting the infodemic when contradictory information emerges. Further, “first line” responders, such as pharmacists, physicians can provide concise, reliable, and accurate information armed with reputable scientific validation to counter misinformation and disinformation. At the same time, one must not forget, additional public awareness and education is effectively improved through the use of strategic media campaigns regarding the thoughtful sourcing and rational use of medications during the pandemic.

## Figures and Tables

**Table 1:** Probability of occurrence of website categories of organic links among first thirty search engine results for “buy ivermectin online” search term.

	Time of Evaluation							
Search Engine Result Page Categories Based on Linked Websites	Dec.20 (*n*, %)		Jan.21 (*n*, %)		Feb.21 (*n*, %)		Mar.21 (*n*, %)	
Online pharmacy websites offering human medicines direct to consumers								
Legitimate online pharmacy	3	10.0%	1	3.3%	3	10.0%	3	10.0%
Illegal medicine seller (rogue online pharmacy)	6	20.0%	5	16.7%	1	3.3%	1	3.3%
Compromised site redirecting to international illegal medicine seller	10	33.3%	6	20.0%	20	66.7%	21	70.0%
Websites offering various ivermectin products for retail use								
Global B2C or B2B e-commerce website	3	10.0%	3	10.0%	2	6.7%	2	6.7%
Veterinary online pharmacy	1	3.3%	1	3.3%	0	0.0%	0	0.0%
Price comparison or intermediary page	2	6.7%	2	6.7%	1	3.3%	0	0.0%
Telemedicine site	1	3.3%	1	3.30%	2	6.7%	1	3.3%
Informative website	2	6.7%	6	20.0%	1	3.3%	1	3.3%
Website not accessible at time of evaluation	2	6.7%	5	16.7%	0	0.0%	1	3.3%
Total	30	100.0%	30	100.0%	30	100.0%	30	100.0%

**Table 2:** Average search engine rank position of organic links.

	Time of Evaluation							
Search Engine Result Page Categories Based on Linked Websites	Dec.20 (rank#, RSD)		Jan.21 (rank#, RSD)		Feb.21 (rank#, RSD)		Mar.21 (rank#, RSD)	
Online pharmacy websites offering human medicines direct to consumers								
Legitimate online pharmacy	18	43.4%	9	0.0%	23.3	13.1%	23,.7	10,.6%
Illegal medicine retailer (rogue online pharmacy)	22	17.0%	22.6	28.1%	28	0.0%	30	0.0%
Compromised site redirecting to international illegal medicine retailer	6.5	46.6%	6	52.7%	11.6	64.5%	12.5	65.4%
Websites offering various ivermectin products for retail use								
Global B2C or B2B e-commerce website	19.7	36.8%	16	45.2%	26	5.4%	26	5.4%
Veterinary online pharmacy	23	0.0%	28	0.0%	-	-	-	-
Price comparison or intermediary page	19.5	3.6%	15.5	4.6%	30	0.0%	-	-
Telemedicine site	12	0.0%	18	0.0%	21	13.5%	23	0.0%
Informative website	25	22.6%	22.3	21.1%	11	0.0%	11	0.0%
Website not accessible at time of evaluation	15.5	132.3%	9.6	97.3%	-	-	16	0.0%

## Data Availability

Datasets, archived SERP content and numerical data supporting reported results can currently be found here: https://drive.google.com/drive/folders/1qLUzE5gvZ2o0-bdYt67_3H8IZjTNZfSZ?usp=sharing (accessed on 16 April 2021). Raw data will be publicly deposited upon request.

## Appendix A

**Table A1:** Potential indicators of patient safety risks of the evaluated online pharmacy websites 1.

Domain URL	Times Appeared in SER Links	Geographical Location of Street Address	Telephone Number Indicated	Primary Phone Number	Health-Care Professional Contact Option	Legit-Script Legitimacy Check	Prescription Requirement	COVID-19 as Health Claim for Ivermectin
1mg.com	1	New Delhi, India	No	-	Yes	certified	Yes	No
1-rx-store.com	1	Prague, Czech Republic	Yes	18007155341	No	rogue	No	No
24x7globalmed.com	2	n.a.	Yes	17184879792 *	No	rogue	No	No
anonympharmacy.com	24	n.a.	Yes	18885247141 **	No	rogue	No	No
blinkhealth.com	4	New York, USA	Yes	18442656444	Yes	certified	Yes	No
blueskydrugs.com	1	British Columbia, Canada	Yes	18669957387	Yes	rogue	Yes	No
canadadrugsonline.com	2	British Columbia, Canada	Yes	18879003784	Yes	rogue	Yes	No
dr-cheap.com	26	n.a.	Yes	18885247141 **	No	rogue	No	No
family24rx.com	2	n.a.	Yes	18882437406 ***	No	rogue	No	No
healthwarehouse.com	4	Florence, KY, USA	Yes	18007487001	Yes	certified	Yes	No
northwestpharmacy.com	3	Langley, BC, Canada	Yes	18665395330	Yes	rogue	Yes	No
order-rxpills.com	2	n.a.	Yes	18882437406 ***	No	rogue	No	No
pharmacity.net	1	n.a.	Yes	18778889761	No	rogue	No	No
reliablerxpharmacy.com	1	n.a.	Yes	18558705513	No	rogue	Yes	No
rx.travelweblog.net(redirect to sale-meds.com)	1	n.a.	Yes	17184879792	No	rogue	No	No
rxindia.com	1	Himachal Pradesh, India	Yes	918628872222	No	Not in database	Yes	Yes
trustnetpharmacy.com	1	n.a.	Yes	18885247141 **	No	rogue	no	No
unitedpharmacies-uk.md	1	-	Yes	441224928494	No	rogue	no	No

Asterisk symbols label the same contact phone number indicating partnership or affiliated networks of online pharmacy websites.
